# Longevity of memory B cells and antibodies, as well as the polarization of effector memory helper T cells, are associated with disease severity in patients with COVID-19 in Bangladesh

**DOI:** 10.3389/fimmu.2022.1052374

**Published:** 2022-12-12

**Authors:** Marjahan Akhtar, Salima Raiyan Basher, Nuder Nower Nizam, Mohammad Kamruzzaman, Fatema Khaton, Hasan Al Banna, M Hasanul Kaisar, Polash Chandra Karmakar, Al Hakim, Afroza Akter, Tasnuva Ahmed, Imam Tauheed, Shaumik Islam, Faisal Ahmmed, Shakil Mahamud, Mohammad Abul Hasnat, Mostafa Aziz Sumon, Asif Rashed, Shuvro Ghosh, Stephen B. Calderwood, Jason B. Harris, Richelle C. Charles, Regina C. LaRocque, Edward T. Ryan, Sayera Banu, Tahmina Shirin, Fahima Chowdhury, Taufiqur Rahman Bhuiyan, Firdausi Qadri

**Affiliations:** ^1^ Infectious Diseases Division, International Centre for Diarrhoeal Disease Research Bangladesh (ICDDRB), Dhaka, Bangladesh; ^2^ Department of Genetic Engineering and Biotechnology, Jagannath University, Dhaka, Bangladesh; ^3^ Department of Cardiology, Department of Oncology, Kurmitola General Hospital, Dhaka, Bangladesh; ^4^ Department of Microbiology, Department of Medicine, Mugda Medical College and Hospital, Dhaka, Bangladesh; ^5^ Division of Infectious Diseases, Massachusetts General Hospital, Boston, MA, United States; ^6^ Departments of Medicine and Pediatrics, Harvard Medical School, Boston, MA, United States; ^7^ Department of Immunology and Infectious Diseases, Harvard T.H. Chan School of Public Health, Boston, MA, United States; ^8^ Institute of Epidemiology, Disease Control and Research (IEDCR), Dhaka, Bangladesh

**Keywords:** COVID-19, asymptomatic, symptomatic, antibodies, memory B cells

## Abstract

The longevity of immune responses induced by different degrees of severe acute respiratory syndrome coronavirus 2 (SARS-CoV-2) infection provides information important to understanding protection against coronavirus disease 2019 (COVID-19). Here, we report the persistence of SARS-CoV-2 spike receptor-binding domain (RBD) specific antibodies and memory B cells recognizing this antigen in sequential samples from patients in Bangladesh with asymptomatic, mild, moderate and severe COVID-19 out to six months following infection. Since the development of long-lived memory B cells, as well as antibody production, is likely to be dependent on T helper (Th) cells, we also investigated the phenotypic changes of Th cells in COVID-19 patients over time following infection. Our results show that patients with moderate to severe COVID-19 mounted significant levels of IgG antibodies out to six months following infection, while patients with asymptomatic or mild disease had significant levels of IgG antibodies out to 3 months following infection, but these then fell more rapidly at 6 months than in patients with higher disease severity. Patients from all severity groups developed circulating memory B cells (MBCs) specific to SARS-CoV-2 spike RBD by 3 months following infection, and these persisted until the last timepoint measured at 6 months. A T helper cell response with an effector memory phenotype was observed following infection in all symptomatic patients, while patients with asymptomatic infection had no significant increases in effector Th1, Th2 and Th17 effector memory cell responses. Our results suggest that the strength and magnitude of antibody and memory B cells induced following SARS-CoV-2 infection depend on the severity of the disease. Polarization of the Th cell response, with an increase in Th effector memory cells, occurs in symptomatic patients by day 7 following infection, with increases seen in Th1, Th2, Th17 and follicular helper T cell subsets.

## Introduction

Coronavirus disease-19 (COVID-19), caused by severe acute respiratory syndrome coronavirus-2 (SARS-CoV-2), is a current global health emergency. Since its discovery in Wuhan, China in December 2019, the SARS-CoV-2 pandemic has caused more than 637 million confirmed COVID-19 cases and about 6.6 million deaths worldwide by November 06, 2022 (1). COVID-19 was first detected in Bangladesh on March 8, 2020 (2). A cross-sectional study conducted in Bangladesh between April 18 and October 12, 2020 reported a 6.4% national prevalence of COVID-19 in the Bangladeshi population (3). Just prior to the current study, the national seroprevalence rate of SARS-CoV-2 specific antibodies was more than 30% in Bangladesh (4). Understanding the nature of immunity following COVID-19 disease is crucial to controlling the pandemic, and provides insight into protection following infection, as well as vaccination and therapeutic interventions. Functional immunity against any infection including SARS-CoV-2 is mediated by long-lived memory T and B cells. Our current understanding of SARS-CoV-2 immunity is mainly based on measurement of antibodies that target the spike and nucleocapsid proteins of SARS-CoV-2. The receptor-binding domain (RBD) portion of the spike S1 protein has been a particularly attractive immunologic target because of its critical role in binding of the virus to the human angiotensin-converting enzyme 2 (ACE2) receptor, which promotes the entry of SARS-CoV-2 into target cells (5–7). Immunological memory following infection or vaccination is primarily mediated by B and T lymphocytes that can bind antigens on viral particles and become activated, replicate, and differentiate rapidly to secrete effector molecules (antibodies or cytokines) to help control infection. Among these effector cells, only approximately 10% can survive and persist as memory cells after clearance of the infection (8). Memory B cells (MBCs) specific for RBD showed kinetics similar to MBCs specific for the whole spike protein during the 8 months after SARS-CoV-2 infection (9). Cross-sectional analysis in patients with COVID-19 revealed that spike-specific MBCs increased over the first approximately 120 days after the onset of COVID-19 symptoms and then plateaued (9). Moreover, longitudinal assessment of individuals recovering from mild COVID-19 showed RBD-specific IgG positive MBCs that persisted until 3 months post-symptom onset and expressed antibodies capable of neutralizing SARS-CoV-2 (10). However, the generation and dynamics of memory B cells has not been determined in patients with a range of COVID-19 disease severity.

T cells, particularly CD4+ T helper (Th) cells, are an indispensable part of adaptive immunity that helps in the development and maintenance of immunological memory. Th cells have been found to maintain the quality of antibody responses, particularly promoting neutralizing antibody responses to provide protective immunity against infection (11). SARS-CoV-2 specific T cells, particularly effector Th cells (T_EM_) and antibody responses persist for up to one year in individuals who experience COVID-19 (12).

However, T cell and antibody responses may not always be completely co-related. For example, asymptomatic COVID-19 patients had low T cell responses although they developed prominent neutralizing antibody responses against the SARS-CoV-2 virus (13). A number of T cell studies following SARS-CoV-2 infection have described the presence of follicular helper T (Tfh), Th1, Th2 and Th17 cells following infection, each one having a characteristic function against infection (14–16). Convalescent individuals recovering from severe COVID-19 showed a higher frequency of spike-specific and functional CXCR3+Tfh cells compared with individuals with mild disease and were positively associated with neutralizing antibody levels (15). Patients with COVID-19 have an increased neutrophil-to-lymphocyte ratio and this has been correlated with induction of Th17 cells (17). The frequency of the different Th cell subsets varies during COVID-19 disease progression. In the acute phase of infection, a higher percentage of senescent PD-1+/ICOS- exhausted Th2 cells were found in patients who died than in those who survived COVID-19 (18). In addition, Th1 polarization with higher cytolytic activity against COVID-19 also correlated with disease pathogenesis. Patients with COVID-19 of varying disease severity had activated Th1 cell phenotypes by the second week following symptom onset (19).

Polarization of the Th cell response seems to play an important role in determining COVID-19 disease severity, although this relationship is still not well understood. Most immunological studies of SARS-CoV-2 responses have been done in symptomatic COVID-19 patients. However, limited information exists for the persistence of SARS-CoV-2 specific memory B cells and the phenotypic responses of T cells in asymptomatic patients compared to symptomatic patients with different disease severity. In the current study, our main objective was to outline the SARS-CoV-2 specific antibody and memory B cell responses in patients with asymptomatic, mild, moderate, and severe COVID-19 disease at different time points out to six months following infection. Moreover, we were also interested in assessing the polarization of phenotypic responses of CD4+Th cells following infection, and its potential association with the severity of infection.

## Methods and materials

### Study design

We conducted a longitudinal cohort study between November 2020 and August 2021 in Dhaka, Bangladesh and enrolled adult patients with COVID-19 (n=100, age ≥18 years), confirmed by SARS-CoV-2 reverse transcription-polymerase chain reaction (RT-PCR) (**Table 1**). Patients were enrolled from Mugda Medical College and Hospital, Dhaka and Kurmitola General Hospital, Dhaka as well as from the community of Mirpur, Dhaka. Disease severity of the patients was classified as asymptomatic, mild, moderate, and severe (n = 25 per group) based on clinical symptoms and oxygen saturation (SpO2) according to WHO guidelines (20), as determined through review of hospital admission records. An asymptomatic case was defined as an individual who was SARS-CoV-2 RT-PCR positive, had no signs or symptoms and who was enrolled from the community (21). Patients with mild disease, enrolled from the hospital and community, had symptoms without having respiratory distress. Patients with moderate disease severity had respiratory distress with SpO2 ≥ 90% and patients with severe disease had respiratory distress with SpO2 <90%; both were enrolled from the hospital. Symptoms found in different categories of disease severity at enrollment are shown in **Table 1**. In addition, we also enrolled adult healthy controls (n=21) who had no history of COVID-19 and were SARS-CoV-2 RT-PCR negative as well as seronegative at enrollment. This study was approved by the Institutional Review Board of the International Centre for Diarrhoeal Disease Research (icddr,b) and the Directorate General of Health Services (DGHS) of Bangladesh. Informed written consent was obtained from all participants at enrollment.

**Table 1:** Demographic information for the study participants.

Parameters	Healthy controlsn=21	COV1D-19 patients with different disease severity			
		Asymptomatic n=25	Mild n=25	Moderate n=25	Severe n=25
Age (years)^a^	42 (31,50)	35 (31,44)	45 (32,53)	50 (38,59)	55 (50,65)
Sex					
Male, n (%)	12 (57)	9 (36)	16 (64)	18 (72)	17 (68)
Female, n (%)	9 (43)	16 (64)	9 (36)	7 (28)	8 (32)
Duration between symptom onset and enrollment^b^	N/A^c^	N/A	9 (6,10)	10 (9,12)	11 (9,13)
SpO2 during hospitalization (%), mean (CI)	N/A	N/A	97.7^d^ (97.0-98.4)	94.0 (92.9-95.2)	82.6 (77.8-87.3)
Symptoms at enrollment, n (%)					
Fever	N/A	N/A	23 (92)	22 (88)	25 (100)
Cough	N/A	N/A	13 (52)	21 (84)	22 (88)
Sore throat	N/A	N/A	3 (12)	1 (4)	3 (12)
Shortness of breath	N/A	N/A	0 (0)	25 (100)	25 (100)
Loss of smell	N/A	N/A	11 (44)	6 (24)	6 (24)
Loss of taste	N/A	N/A	15 (60)	9 (36)	9 (36)
Runny nose	N/A	N/A	4 (16)	4 (16)	1 (4)
Chest pain	N/A	N/A	2 (8)	2 (8)	1 (4)
Muscle aches	N/A	N/A	3 (12)	4 (16)	5 (20)
Joint pain	N/A	N/A	4 (16)	5 (20)	1 (4)
Headache	N/A	N/A	8 (32)	7 (28)	2 (8)
Vomiting	N/A	N/A	3 (12)	1 (4)	2 (8)
Diarrhea	N/A	N/A	2 (8)	5 (20)	3 (12)
Other^e^	N/A	N/A	7 (28)	1 (4)	2 (8)

a Mean with 95% confidence interval (CI).

b Median number of days (interquartile range, IQR).

c N/A; not applicable.

d 19 out of 25 patients were hospitalized as they were unable to isolate at home.

e Other symptoms included fatigue, malaise, nausea, back pain, generalized weakness, hiccups, sneezing and tonsillitis.

### Sample collection and processing

We collected blood and nasopharyngeal swabs (NPS) from all patients at enrollment (day 1) and again on day 7, day 28, day 90 (3 months) and day 180 (6 months) after enrollment. From healthy controls, blood and NPS specimens were collected once on enrollment. Serum was separated from whole blood after centrifugation of the tubes at 700x *g* for 15 minutes and kept frozen (-80 °C) until the time of laboratory analysis. Peripheral blood mononuclear cells (PBMCs) were separated by density-gradient centrifugation using Ficoll-Paque PLUS (Cytiva, USA). After isolation, PBMCs were cryopreserved using the cryoprotectant (90% FBS, 10% DMSO). These cryopreserved cells were later used in the MBC assay and for flow cytometrical analysis of T cells and other immunological assays reported (22).

### Enzyme-linked immunosorbent assay

Serum specimens were collected at all time points from each patient and once from healthy controls, and were used to determine IgG antibodies specific to spike RBD of SARS-CoV-2 (Wuhan strain) using an ELISA as described previously (4, 23). Briefly, 96 well Nunc^®^ MaxiSorp™ plates (ThermoFisher) were coated with 100 µL of SARS CoV-2 RBD antigen (1μg/ml carbonate buffer) and incubated for 1 hour at room temperature (RT). Plates were blocked for 30 minutes at RT with 300 µL of 5% nonfat milk in phosphate-buffered saline (PBS). Heat-inactivated serum samples (serially diluted 1:100, 1:400, 1:1600 and 1:6400 in 5% Milk- 1X PBS 0.05% Tween) were added to the plate (100 μL/well) and incubated for 1 hour at 37°C. A specific monoclonal antibody to RBD of known concentration (CR3022) was added to the plate, eight 2-fold serial dilutions were performed starting at 25 ng/ml. Subsequently, goat anti-human IgG horseradish peroxidase-conjugated secondary antibodies (Jackson ImmunoResearch) diluted 1:5000 in 5% milk in PBST were added to the plates (100 μL/well) and incubated at ambient temperature for 30 min. Bound secondary antibodies were detected using ortho phenylenediamine (OPD; Sigma, 200 μL/well) in 0.1 M sodium citrate buffer (pH 4.5) and 30% H_2_O_2_. Plates were incubated at RT for 20 minutes in the dark. Optical density (OD) was measured at 450 nm and 570 nm in the Eon (Biotek) ELISA Reader; OD values were adjusted by subtracting the 570 nm OD nm from the 450 nm OD.

### Memory B cell assay

We determined RBD-specific IgG+ memory B-cells using an enzyme-linked immunosorbent spot (ELISPOT) assay. Cryopreserved PBMCs from healthy controls (n=10) and patients of all disease categories from days 1, 28, 90 and 180 (n=8-13/day from each group) were thawed and rested, at a concentration of 10^6^ cells/mL, in RPMI complete medium containing 10% FBS (Invitrogen), 1% Penicillin-Streptomycin (Invitrogen), 1% Sodium-Pyruvate (Invitrogen), 1% L-Glutamine (Invitrogen) and 1% Gentamicin (Life Technology) for 18 hours in 37°C in 5% CO_2_. After incubation, cells were stimulated at a concentration of 5 x 10^5^/mL in each well, and expanded as described previously (24) in a 24-well tissue culture plate (Falcon) with a stimulation media containing 6 µg/ml CpG oligonucleotide (*In vivo*gen), a 1/100,000 dilution of crude pokeweed mitogen extract, and a 0.1µg/ml *Staphylococcus aureus* Cowan (Sigma) at 37°C in 5% CO_2_ for 6 days. After polyclonal stimulation, the cells were harvested, thoroughly washed and counted to be seeded onto nitrocellulose bottom plates (MAHA S4510; Millipore). To measure the total IgG secreting MBC, 2 x 10^4^ cells were seeded onto monoclonal anti-human IgG capture antibody (5µg/mL, Jackson Immunoresearch) coated duplicate wells and diluted serially (2-fold) from row A to G. For measuring the RBD specific IgG secreting MBC, upto 10^6^ cells were seeded in a single well or duplicate wells, coated with RBD (SARS-CoV-2 Wuhan strain, a gift from A. Schmidt lab, Ragon Institute, Boston MA) at a concentration of 5 µg/mL. The indicated coating concentration of RBD antigen was found to provide maximal number of MBC spots in initial set-up experiments testing a range of different concentrations. As negative control, cells were seeded onto PBS coated wells. Plates were then incubated at 37°C in 5% CO_2_ for 5 hours with goat anti-human IgG horseradish peroxidase-conjugated secondary antibodies (1:1000, Jackson ImmunoResearch). IgG spots were visualized with substrate 3-amino-9-ethylcarbazole (AEC) substrate (Calbiochem, MA) as red spots. Spots were counted using an automated ELISPOT reader (Immunospot 5.1, Cellular Technologies Ltd, OH), considering that minimum area of each Spot Forming Cells (SFC) is 0.0051mm^2^ (**Supplementary Figure 1A**). Total and antigen-specific MBC spots were calculated as IgG SFC/10^6^ cells. Frequencies of MBCs against RBD are expressed as the percentage of specific MBCs per total IgG MBCs at that time point.

### Flow cytometric analysis

Cryopreserved PBMCs isolated from healthy controls (n=10) and day 1, day 7, day 28 and day 180 from patients (n=5-10/time point/disease category) were thawed, rested and stained with LIVE/DEAD™ Fixable Near-IR Dead Cell Stain Kit (Thermo Fisher Scientific) to exclude dead cells. To analyze the frequency of different Th cell subsets, PBMCs were stained with anti-CD3-AmCyan (clone SK7, BD), anti-CD4-APC (clone SK3, BD), anti-CD45RO- PerCP/Cyanine5.5 (Biolegend), anti-CCR7-PECy7 (Biolegend), anti-CXCR5-AF488 (RF8B2, BD Pharmingen), anti-CXCR3-PE (1C6/CXCR3, BD), anti-CCR6-BV421 (11A9, BD Horizon), anti-PD-1- PE-CF594 (EH12.1, BD) and anti-ICOS- BV605 (DX29, BD). Among the lymphocytes, 95% of cells were found to be alive as determined by live/dead staining. Different subsets of live T cells were analyzed following the gating strategy shown in **Supplementary Figure 2**.

### Statistical analysis

Statistical analyses were performed using GraphPad Prism 6 (GraphPad Software, Inc., La Jolla, CA). Flow cytometry figures were generated using FlowJo software (version 10.0). Memory B cells were enumerated using an automated ELISPOT reader with aid of the Immunospot software version 5.1 (Cellular Technologies Ltd). The Mann-Whitney test was used to evaluate immunological differences between patients with COVID-19 and healthy individuals. *P* values of <0.05 were considered significant. IgG antibody levels ≥500 ng/ml were considered seropositive (4, 23).

## Results

### SARS-CoV-2-specific serum IgG antibodies

**Figure 1:**
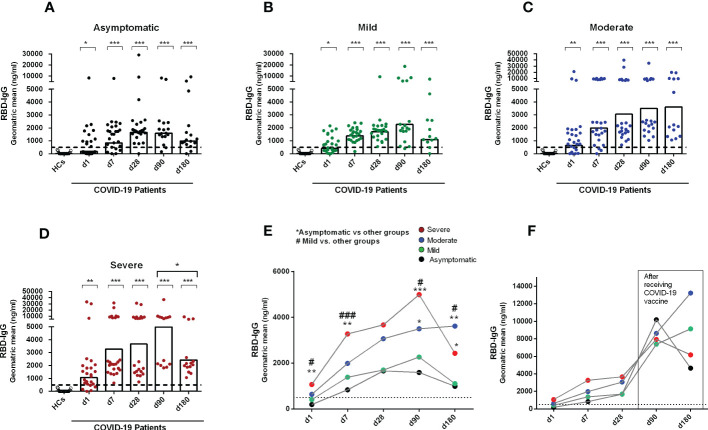
Antibody responses in COVID-19 patients with different disease severity, compared to healthy controls. RBD specific IgG was analyzed in serum samples collected from healthy adults (n=21) as well as patients with **(A)** asymptomatic (n = 25), **(B)** mild (n = 25), **(C)** moderate (n = 25), and **(D)** severe (n = 25) COVID-19 on day 1, 7, 28, 90 and 180. Each symbol represents one individual, and bars indicate geometric mean values. Statistical analysis was performed between healthy controls and different time points in patients using the Mann-Whitney U test. Compared to healthy controls, all patients had significantly higher (P < 0.0001) IgG antibodies at all time points. **(E)** Line graphs showing the geometric mean values of antibody concentration in patients with differing disease severity. Statistical analysis was performed between asymptomatic vs. other groups (*) and mild vs. other groups (#) on each day using the Mann-Whitney U test. **(F)** line graphs showing the geometric mean values of antibody concentrations on day 90 (asymptomatic, n = 7; mild, n = 4; moderate, n = 4; severe, n = 6) and 180 (asymptomatic, n = 8; mild, n = 9; moderate, n = 10; severe, n = 6) in a patient group after receiving a COVID-19 vaccine one month after infection. Dotted line indicates the cut-off (500 ng/ml) limit of seropositivity. ^#/*^ *P <*0.05, ^##/**^ *P <*0.01, ^###/***^ *P <*0.001, ns, not significant; *P >*0.05.

We first performed a longitudinal analysis of circulating anti-SARS-CoV-2 serum antibodies. A few of the recovered patients received a COVID-19 vaccine (Covishield, Moderna, Pfizer, or Sinopharm) one month after COVID positivity and their results were therefore excluded at day 90 and day 180 from the analysis of antibody responses conferred by natural infection (**Figures 1A-E**). While anti-SARS-CoV-2 RBD-specific IgG antibodies were undetectable in blood from healthy controls, patients with COVID-19 had detectable serum IgG to RBD already on the day of enrollment in asymptomatic and symptomatic patients with COVID-19. As previously reported from our group (23), compared to healthy individuals, patients with COVID-19 with different severity status had significantly increased levels of IgG antibodies out to six months after COVID positivity (P<0.05, **Figures 1A-D**).

Asymptomatic patients developed peak IgG antibody responses by one month (d28) after COVID-19 positivity, which was stable until 3 months of infection. However, by six months after COVID positivity, antibody levels decreased in asymptomatic patients (**Figures 1A, E**). Patients with mild COVID-19 also showed similar antibody kinetics as asymptomatic individuals; however, in that group, the peak antibody level was found at 3 months after COVID positivity (**Figures 1B, E**). Patients with moderate and severe COVID-19 had different antibody kinetics compared to asymptomatic and mild patients (**Figures 1C, D**). Antibody concentration was increased in those groups out to six months (day 180) after COVID-19 positivity, and compared to the groups with lesser severity, these groups with higher severity had significantly higher IgG responses to RBD on day 180 (**Figure 1E**). We also analyzed antibody responses in a few patients who received a COVID-19 vaccine 1 month after infection (**Figure 1F**), and as expected, these individuals had a substantial boost in antibody titers out to days 90 and 180 following initial infection.

### Memory B cell responses in patients with COVID-19

**Figure 2:**
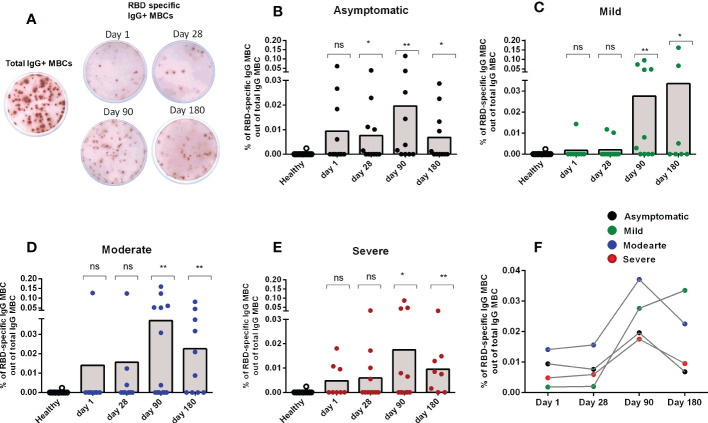
Memory B cell responses in COVID-19 patients with differing disease severity, compared to healthy controls. **(A)** Representative image of total and RBD-specific IgG+ MBC spots. Frequency of RBD specific IgG+ MBC within the total IgG+ MBCs from healthy adults (n = 10), as well as patients with **(B)** asymptomatic (n = 10-11), **(C)** mild (n = 8-11), **(D)** moderate (n = 9-13), and **(E)** severe (n = 8-11) COVID-19 on day 1, 28, 90 and 180. Each symbol represents one individual, and bars indicate mean values. Statistical analysis was performed between healthy controls and different time points in patients using the Mann-Whitney U test. **(F)** Line graphs showing mean values of the frequency of RBD specific IgG+ MBC within the total IgG+ MBCs in patients with differing severity of infection. No significant difference in the frequency of MBCs were found between the groups with differing severity of infection. Total and antigen-specific MBC spots were calculated as IgG spots/10^6^ cells. Frequencies of MBCs against RBD are expressed as the percentages of specific MBCs per total IgG MBCs. ^*^ *P <*0.05, ^**^ *P <*0.01, ^***^ *P <*0.001, ns, not significant; *P >*0.05.

After studying the levels of RBD specific IgG antibodies, the next step was to determine whether RBD-specific IgG+ MBCs were found following infection in patients with different degrees of disease severity. No SARS-CoV-2 specific IgG MBCs were detected in healthy controls. Following infection, RBD-specific IgG MBCs were found by day 90 in patients in all categories of disease severity, and these persisted until the last time point examined (day 180 after COVID positivity) (**Figures 2A-E**). Patients with mild and moderate disease severity had the highest levels of RBD-specific IgG MBCs at day 180 following infection (**Figure 2F**).

### Kinetics of peripheral blood CD4+ memory Th cells in patients with COVID-19

**Figure 3:**
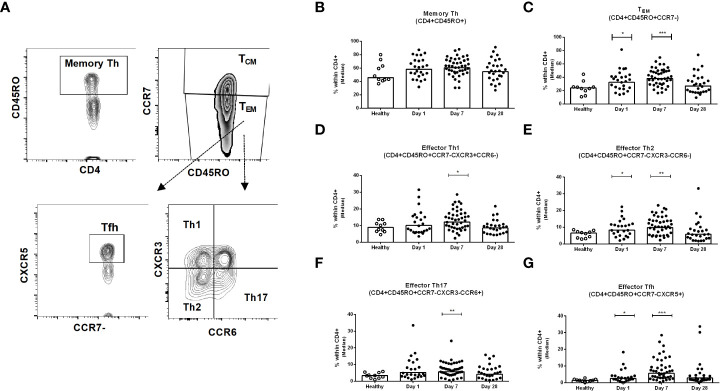
Kinetics of peripheral blood CD4+ memory Th cells, effector memory Th cells, and effector Th1, Th2, Th17 and Tfh cells in COVID-19 patients and healthy controls. **(A)** Representative images of the gating strategy of Th cells analyzed using flow cytometry. Frequencies of **(B)** memory Th (CD4+CD45RO+) **(C)** effector memory TEM (CD4+CD45RO+CCR7-), **(D)** effector memory Th1 (CD4+CD45RO+CCR7-CXCR3+CCR6-), **(E)** effector memory Th2 (CD4+CD45RO+CCR7-CXCR3-CCR6-), **(F)** effector memory Th17 (CD4+CD45RO+CCR7-CXCR3-CCR6+), and **(G)** effector memory Tfh (CD4+CD45RO+CCR7-CXCR5+) populations within CD4+ cells in PBMCs isolated from healthy adults (n = 10) and patients with COVID-19 on days 1 (n = 24), 7 (n = 45), and 28 (n = 26). Each symbol represents the percentage of cells for one individual, and bars indicate median values. Statistical analysis was performed between healthy controls and different time points in patients using Mann-Whitney U test. *P < 0.05, **P < 0.01, ***P < 0.001.

We investigated the frequencies of different sub-populations of Th cells in PBMC samples collected from healthy and patients with COVID-19 on day 1, day 7 and day 28 following infection (**Figure 3A**). The frequencies of memory Th cells (CD3+CD4+CD45RO+) among CD4+ helper T cells after infection did not differ significantly from levels observed in healthy controls, although a trend for increased frequencies was observed on day 7 (**Figure 3B**). There are two types of memory T cells in the circulation, central (T_CM_) and effector (T_EM_) memory T cells. The proportions of effector memory T_EM_ cells (CD45RO+CCR7-) were increased significantly on day 1 and day 7 after COVID-19 positivity, compared to healthy controls (P<0.05, **Figure 3C**); the proportions of T_EM_ cells then decreased on day 28 and a majority of patients had comparable levels as the controls by this time point. In contrast, compared to healthy controls, a lower number of central memory T_CM_ cells was observed on day 7 in the patients (data not shown). We next determined the phenotypic responses of T_EM_ cell subsets in patients, defined by surface expression of CXCR3, CCR6, and CXCR5, and compared them with healthy controls. Following COVID-19, patients had increased levels of effector memory Th1, Th2, Th17 and Tfh cells that peaked on day 7 following infection and then largely returned to baseline by day 28 (**Figures 3D-G**). Effector Tfh cells can be further divided based on CXCR3 and CCR6 homing markers. We determined the frequency of effector Tfh cells with Th1, Th2 and Th17 phenotypes. Compared to healthy individuals, significantly increased levels of Tfh cells with Th1, Th2 and Th17 phenotypes were observed in COVID-19 patients on day 7 (**Supplementary Figure 3**).

### T_EM_ cell subsets in patients with different disease severity

**Figure 4:**
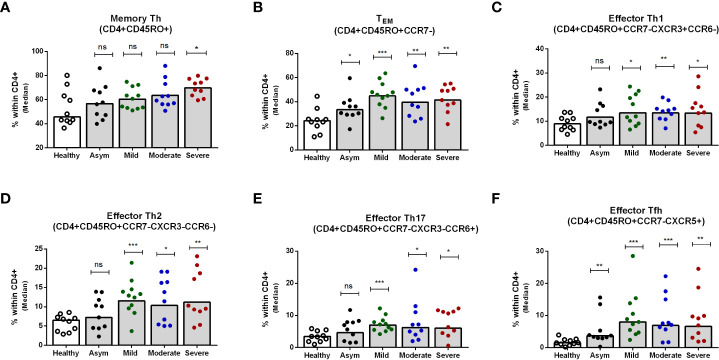
Frequencies of peripheral blood CD4+ memory Th cells, effector memory Th cells, and effector Th1, Th2, Th17 and Tfh cells in patients with differing disease severity of COVID-19, compared to healthy controls. Frequencies of **(A)** memory Th (CD4+CD45RO+), **(B)** effector memory TEM (CD4+CD45RO+CCR7-), **(C)** effector memory Th1 (CD4+CD45RO+CCR7-CXCR3+CCR6-), **(D)** effector memory Th2 (CD4+CD45RO+CCR7-CXCR3-CCR6-), **(E)** effector memory Th17 (CD4+CD45RO+CCR7-CXCR3-CCR6+), and **(F)** effector memory Tfh (CD4+CD45RO+CCR7-CXCR5+) populations within CD4+ cells in PBMCs isolated from healthy adults (n = 10) and patients with asymptomatic, mild, moderate and severe COVID-19 on day 7 (n = 10-11 per group). Each symbol represents the percentage of cells for one individual, and bars indicate median values. Statistical analysis was performed between healthy controls and patients using Mann-Whitney U test. *P < 0.05, **P < 0.01, ***P < .001.

Next, we determined the memory Th cell subsets in patients with asymptomatic, mild, moderate and severe COVID-19 on day 7 after enrollment, since the changes seen peaked at that time point. Hospitalized patients with severe COVID-19 disease had significantly higher memory Th cells overall compared to healthy individuals, although this was not seen in patients with asymptomatic, mild or moderate COVID-19 (**Figure 4A**). Effector memory Th cells increased significantly in all levels of disease severity compared to healthy controls (**Figure 4B**). Within the effector memory subset, all symptomatic patients with mild, moderate or severe COVID-19 disease had significantly higher levels of effector Th1, Th2, Th17 and Tfh cells compared to healthy controls, while asymptomatic patients had a significantly increased frequency only of the effector Tfh subset (P<0.05, **Figures 4C-F**). Analysis of effector memory Th cells on day 180 of patients showed comparable levels of whole memory as well as effector memory Th cells in patients and controls (**Supplementary Figures 4A, B**). However, compared to the controls, significantly increased frequencies of effector Tfh was observed on day 180 in all disease categories of patients. Effector Th2 was found higher in asymptomatic and moderate patients (**Supplementary Figures 4D, F**).

### Activated and exhausted Th cells in COVID-19 patients

**Figure 5:**
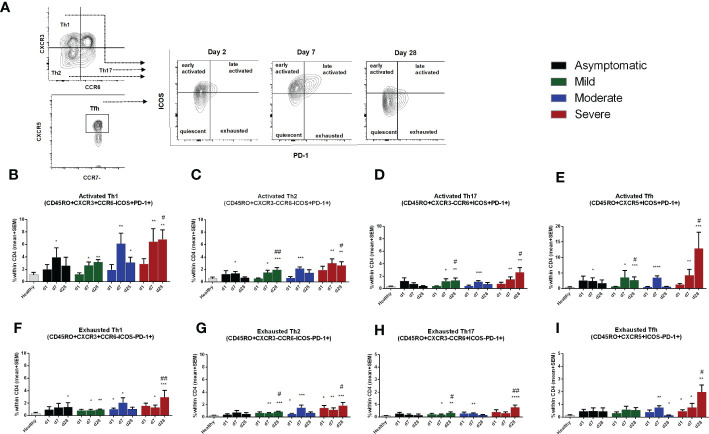
Frequencies of activated and exhausted Th cells in patients with differing severity of COVID-19 infection compared to healthy controls. **(A)** Representative images of different stages of Th cell activation analyzed using flow cytometry. Frequencies of ICOS+PD-1+ late activated memory **(B)**, Th1 **(C)**. Th2 **(D)**. Th17. and **(E)** Tfh, as well as frequencies of ICOS-PD-1+ exhausted memory **(F)**, Th1 **(G)**, Th2 **(H)**, Th17, and **(I)** Tfh populations within CD4+ cells in PBMCs isolated from healthy adults (n =1 0) and patients with asymptomatic, mild, moderate and severe COVID-19 on day 1 (n = 6-7 per group), 7 (n = 10-12 per group) and 30 (n = 8-10 per group). Bars indicate mean+SEM values. Asterisks (*) represent significant differences between healthy controls vs. asymptomatic, mild, moderate and severe patients and hash (#) represent significant differences between asymptomatic patients vs. mild, moderate and severe patients on different days. Statistical analysis was performed between healthy controls and patients using Mann-Whitney U test. *P < 0.05, **P < 0.01, ***P < 0.001.

We evaluated different activation and exhaustion stages of the CD45RO+ memory Th cell subsets based on the expression of PD-1 and ICOS markers (**Figure 5A**). Cells without surface expression of PD-1 or ICOS were considered as quiescent Th cells; PD-1-, ICOS+ cells as early-activated cells, PD-1+, ICOS+ as late activated cells; and PD-1+, ICOS- as exhausted cells (18). Compared to healthy controls, no significant changes in the percentages of quiescent and early activated Th cells occurred in COVID-19 patients from day 1 to day 28 after infection. However, late activated and exhausted Th cell subsets increased in the 28 days following infection (**Supplementary Figure 5**). Compared to healthy controls, patients with asymptomatic COVID-19 had significantly higher percentages of PD-1+, ICOS+ activated Th1, Th2 and Tfh cell subsets on day 7 after infection, which returned to the levels seen in healthy controls by day 28. On the other hand, symptomatic patients with COVID-19 had increased levels of activated Th1, Th2, Th17 and Tfh cell subsets on both day 7 and day 28 following infection. Within the disease categories, patients with severe illness had the highest frequency of activated Th cells (**Figures 5B-E**). After activation and proliferation, T cells also exhibit an exhausted phenotype, which is a state of dysfunction with reduced secretion of cytokines (25). We observed that symptomatic patients, but not asymptomatic patients, had significantly elevated levels of exhausted T cells on both day 7 and day 28 compared to healthy controls (**Figures 5F-I**). As for the activated phenotype, patients with severe COVD-19 had the most abundant exhausted Th cells in the circulation., particularly on day 28.

## Discussion

A thorough understanding of immunological parameters including humoral and cell-mediated components is critical to gaining insights into the role of natural SARS-CoV-2 infection in providing protective immunity as well as for designing an effective vaccine to eradicate the current pandemic. This study sought to determine whether asymptomatic and symptomatic SARS-CoV-2 infection in patients with mild, moderate and severe disease patterns induced CD4+ helper T cells as well as antigen-specific memory B cells and antibody responses out until six months following COVID-19 infection. Our group has shown RBD-specific antibody responses increase rapidly in COVID-19 patients and the magnitude of the responses depend on the severity of the disease course (23). However, in addition to circulating antibody responses, assessment of longevity of SARS-CoV-2-specific memory B cells (MBCs) in patients with different disease severity is important to understanding protective immunity from a subsequent infection. In this study, we show that patients recovered from COVID-19 had serum anti-spike RBD IgG antibodies out until six months after the infection was identified; however, the magnitude of the immune response depended on disease severity (23). We also showed that patients with moderate and severe disease, who required hospitalization as well as had respiratory distress, had significantly higher antibody levels compared to mild or asymptomatic patients. All observed immunological responses in these patients might be against the SARS-CoV-2 Beta (B.1.351) and Delta (B.1.617) variants since during the study period, these were the main circulating SARS-CoV-2 variants in the study population (26).

Patients with moderate to severe COVID-19 had more durable antibody responses than those with mild or asymptomatic infection. Patients at either end of the disease spectrum (mild or severe illness) had antibody responses that peaked at 3 months after infection and then declined at 6 months, while the magnitude of IgG responses was more stable in patients with moderate disease and did not decline until six months. Patients with asymptomatic infection had their highest responses at day 28 after infection, and these slowly decreased by 6 months. Our results are consistent with previous findings that patients with mild COVID-19 have reduced levels of anti-SARS-CoV-2 spike antibodies at 3 to 4 months after infection (27).

SARS-CoV-2 infection elicits IgG memory B cells against spike RBD in patients with all disease severities. As shown in other studies (9, 28, 29), our findings also demonstrate that SARS-CoV-2-specific memory cells develop by 90 days after COVID-19 infection. Flow cytometric analyses have shown that during the acute phase of COVID-19 infection, RBD-specific MBCs primarily exist as non-class switched forms expressing IgM and IgD (30), and to a lesser extent, IgG+ class-switched memory cells (30, 31). Similarly, using an ELISPOT assay, we also found a small frequency of IgG+ MBCs on enrollment in patients with severe COVID using an ELISPOT assay. The frequency and longevity of spike RBD-specific memory cells can vary widely depending on the disease condition and the individual characteristics of the immune system. Most of the patients had a detectable level of IgG+ memory B cells at 3 months after infection. Most patients of the asymptomatic and mild groups did not generate memory B cell responses on day 90 and day 180 as observed in moderate and severe patients. We speculate that in addition to RBD, memory B cells pools of COVID-19 patients might be directed against other prominent antigens of SARS-CoV-2, e.g. spike protein, nucleocapsid protein etc. Moreover, the RBD protein we used in the Memory B cell assay was from Wuhan variant of SARS-CoV-2. We might get more robust responses if we could use RBD from other variants (beta, delta, etc) of SARS-CoV-2 in our current assay. We have not studied IgM+ and IgA+ memory B cells since it has been recently reported that IgG+ memory B cells were more prominent and stable, compared to short-lived IgM and less frequent IgA MBCs after SARS-CoV-2 infection (9). Consistent with our findings, other studies have also shown a higher frequency of RBD-specific IgG+ memory B cells in patients with COVID-19 ninety days after symptom onset (9, 10). The breadth and longevity of MBCs are particularly important for mounting rapid recall responses to subsequent antigen exposure since it is known from other viral infections that recalled IgG+ memory B cells tend to rapidly differentiate into plasma cells without re-entering a germinal center and provide durable long-term immunity (32, 33).

Generation and maintenance of immunological memory are unique properties of adaptive immune responses and require the presence of T helper cells to provide help to B cells to clonally expand, to establish germinal centers, to develop class-switched and affinity-matured antigen receptors as well as to differentiate into effector antibody-secreting plasmablasts that protect the host from pathogens. T cells may have a prominent role in SARS-CoV-2 infection as seen in other respiratory viral infections including human influenza and HIV (32, 34), and T cell activation is associated with increased disease severity (35). Interestingly, we have seen that only patients with severe COVID-19 disease developed CD45RO+ memory Th cells in the acute phase of infection. However, in all symptomatic patients, we observed that phenotypic changes occur within the effector T_EM_ subsets. T_EM_ cells are distinguished by their ability to express immediate effector functions by producing cytokines, and they can rapidly migrate to peripheral tissues for the elimination of infection (36). In contrast to another study (37), our data suggest that in patients with mild to severe COVID-19, central memory Th cells decrease in the setting of increased effector phenotypes in all Th cell subsets, including Tfh, Th1, Th2 and Th17 cells. In contrast, Th1, Th2, and Th17 effector cells did not increase in asymptomatic patients. The fewer phenotypic Th cells in asymptomatic patients was matched by the lower magnitude of antibody responses in these patients. However, a few studies have suggested that asymptomatic individuals produce a detectable level of SARS-CoV-2-specific T cells (38), although inferences on the immunity conferred by asymptomatic infection are hampered by significant methodological variations in the studies as well as knowledge of the true-onset of infection. Here, one of our limitations was that we could not analyze T cell responses to specific antigens of SARS-CoV-2; nevertheless, previous studies have shown that spike S1-specific circulating T_EM_ cells retain functional responsiveness and exhibit improved effector capacities such as activation, proliferation, and secretion of cytokines (37, 39). In contrast, the frequency of effector follicular Tfh cells was induced in patients with COVID-19 irrespective of the severity of infection. In viral infections, Tfh cells are crucial to induction of high-affinity antibodies capable of neutralizing the pathogen (14). In addition, during SARS-CoV-2 infection, the numbers of RBD-specific IgG+ MBCs were found to be significantly correlated with circulating follicular helper T cell numbers (40). There is evidence that the type of helper T cells impacts the disease outcome of infection, including during SARS-CoV-2 infection, by the expression of activation or exhaustion markers (18, 41, 42). In SARS-CoV-2 infection, Th1 mediated immune responses during active SARS-CoV-2 infection were associated with a better prognosis and resolution of COVID-19, while an increased percentage of senescent Th2 cell responses was found in patients who died from COVID-19 (18).

In conclusion, we demonstrate that RBD-specific IgG memory B cells are present and peak by three months after SARS-CoV-2 infection, with a subsequent decrease by 6 months. We also found that T_EM_ cells play an important role in patients with mild to severe disease and the activation process of these cells is associated with disease outcome. MBC responses paralleled the longevity and magnitude of anti-SARS-CoV-2 antibodies in our population; higher responses were found in patients with moderate and severe disease compared to those with mild and asymptomatic infection. However, the functional capacity of these antibodies needs to be determined in future studies.

## Data availability statement

The raw data supporting the conclusions of this article will be made available by the authors, without undue reservation.

## Ethics statement

The studies involving human participants were reviewed and approved by Institutional Review Board of the International Centre for Diarrhoeal Disease Research (icddr,b) and the Directorate General of Health Services (DGHS) of Bangladesh. The patients/participants provided their written informed consent to participate in this study.

## Author contributions

FQ, TB, FC, designed the study. AA, TA, IT performed clinical analysis. MA, SRB, NN, MK, FK, SM, HB, MHK, PK, AH, SI performed the laboratory work and immunological analyses. MA, SRB, NN, MK analyzed the data. MA and TB planned and drafted the manuscript. FQ, TB, SB, SC, RC, ER FC, TS, JBH, RL reviewed the manuscript. All authors contributed to the article and approved the submitted version.
