# Supplementary material for: Longevity of memory B cells and antibodies, as well as the polarization of effector memory helper T cells, are associated with disease severity in patients with COVID-19 in Bangladesh
Source: Front Immunol. 2022 Dec 12;13:1052374. doi: 10.3389/fimmu.2022.1052374 (PMC9791541; doi:10.3389/fimmu.2022.1052374)
Supplement: Supplementary file 1 [file Presentation_1.pptx]

## Slide 1
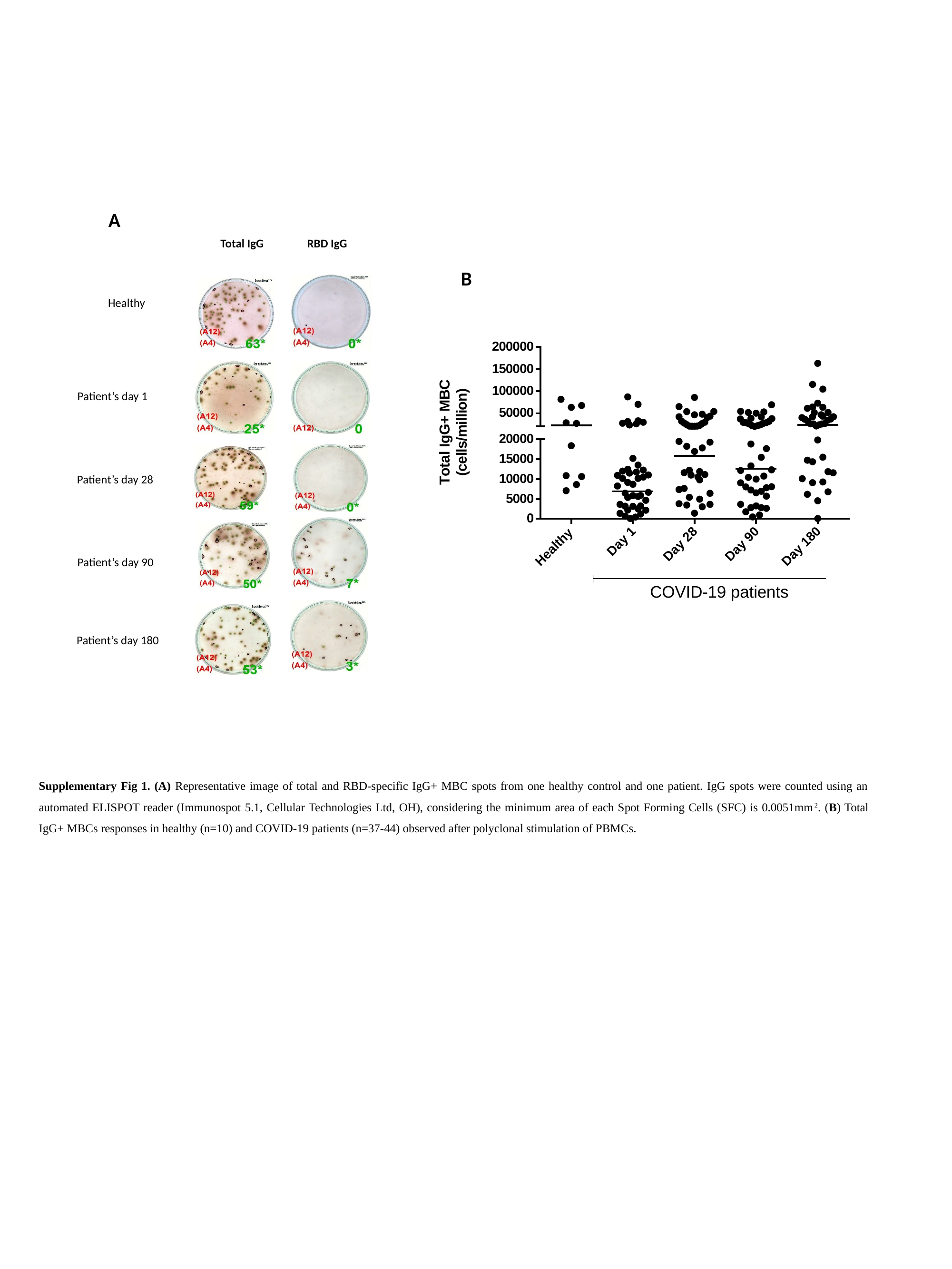

A
Total IgG RBD IgG
Healthy
Patient’s day 1
Patient’s day 28
Patient’s day 90
Patient’s day 180
B
Supplementary Fig 1. (A) Representative image of total and RBD-specific IgG+ MBC spots from one healthy control and one patient. IgG spots were counted using an automated ELISPOT reader (Immunospot 5.1, Cellular Technologies Ltd, OH), considering the minimum area of each Spot Forming Cells (SFC) is 0.0051mm2. (B) Total IgG+ MBCs responses in healthy (n=10) and COVID-19 patients (n=37-44) observed after polyclonal stimulation of PBMCs.

## Slide 2
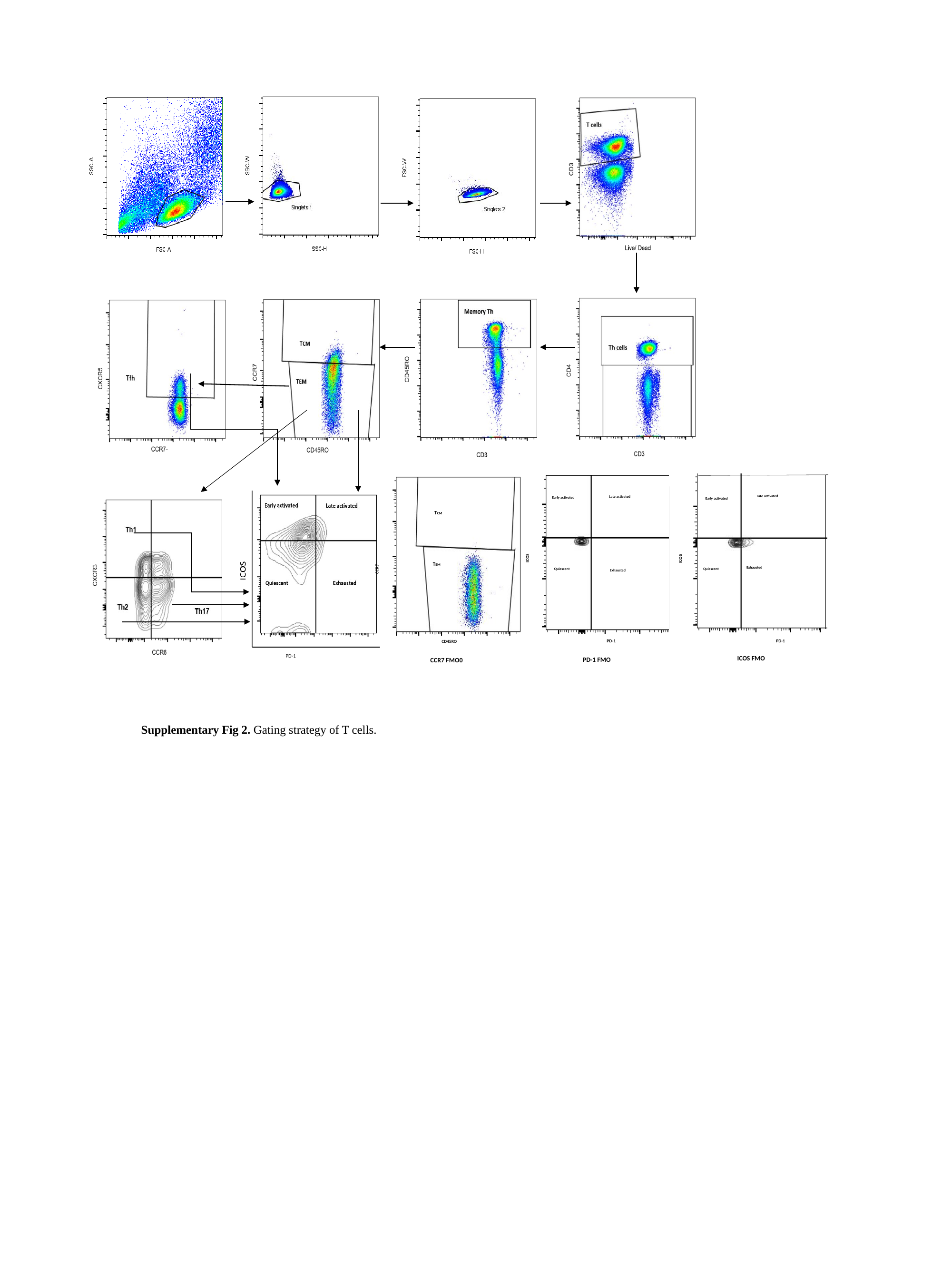

ICOS
ICOS
CCR7
Late activated
Late activated
Early activated
Early activated
TCM
TEM
Exhausted
Quiescent
Quiescent
Exhausted
PD-1
PD-1
CD45RO
ICOS FMO
PD-1 FMO
CCR7 FMO0
PD-1
Supplementary Fig 2. Gating strategy of T cells.

## Slide 3
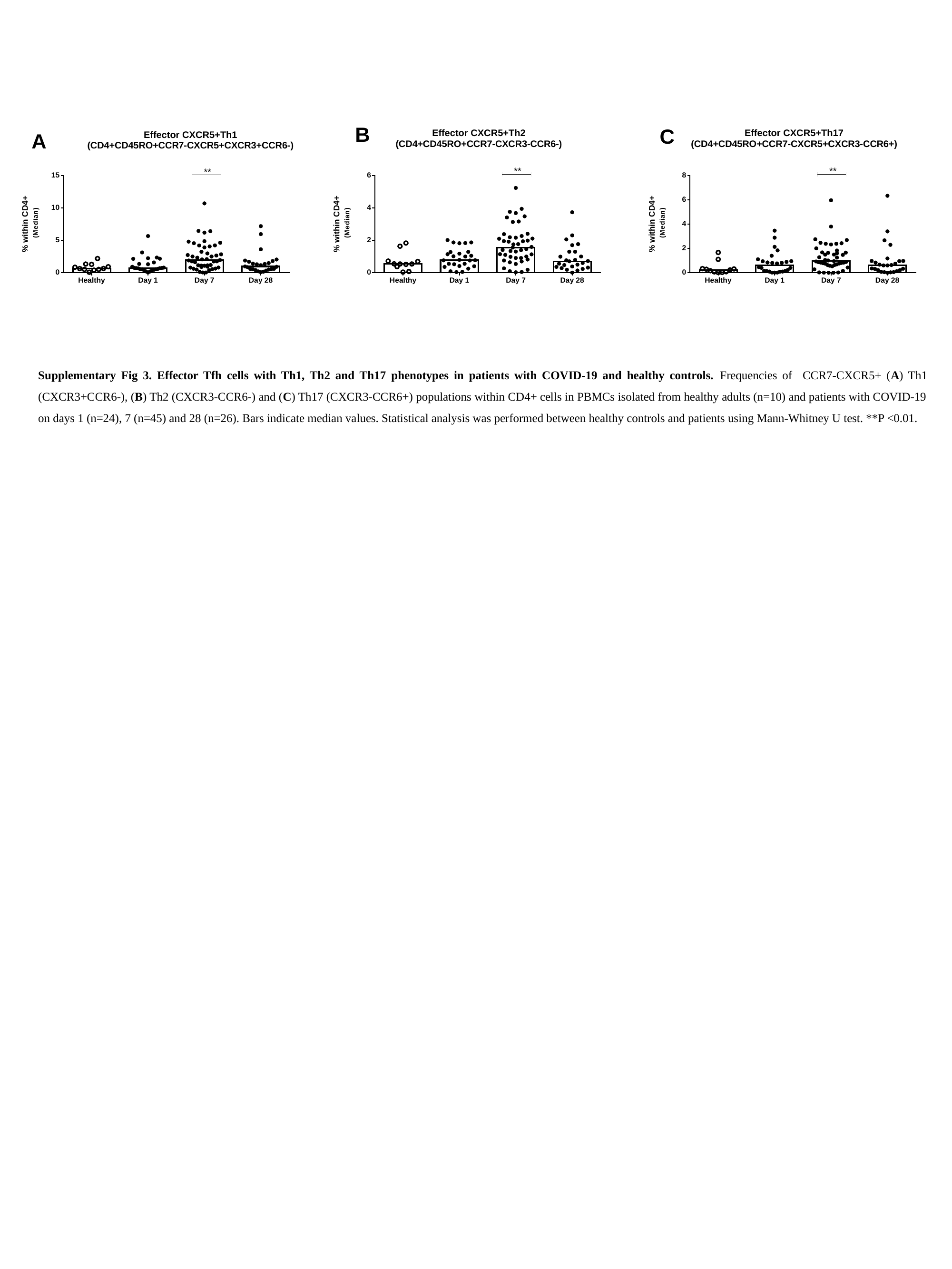

Supplementary Fig 3. Effector Tfh cells with Th1, Th2 and Th17 phenotypes in patients with COVID-19 and healthy controls. Frequencies of CCR7-CXCR5+ (A) Th1 (CXCR3+CCR6-), (B) Th2 (CXCR3-CCR6-) and (C) Th17 (CXCR3-CCR6+) populations within CD4+ cells in PBMCs isolated from healthy adults (n=10) and patients with COVID-19 on days 1 (n=24), 7 (n=45) and 28 (n=26). Bars indicate median values. Statistical analysis was performed between healthy controls and patients using Mann-Whitney U test. **P <0.01.

## Slide 4
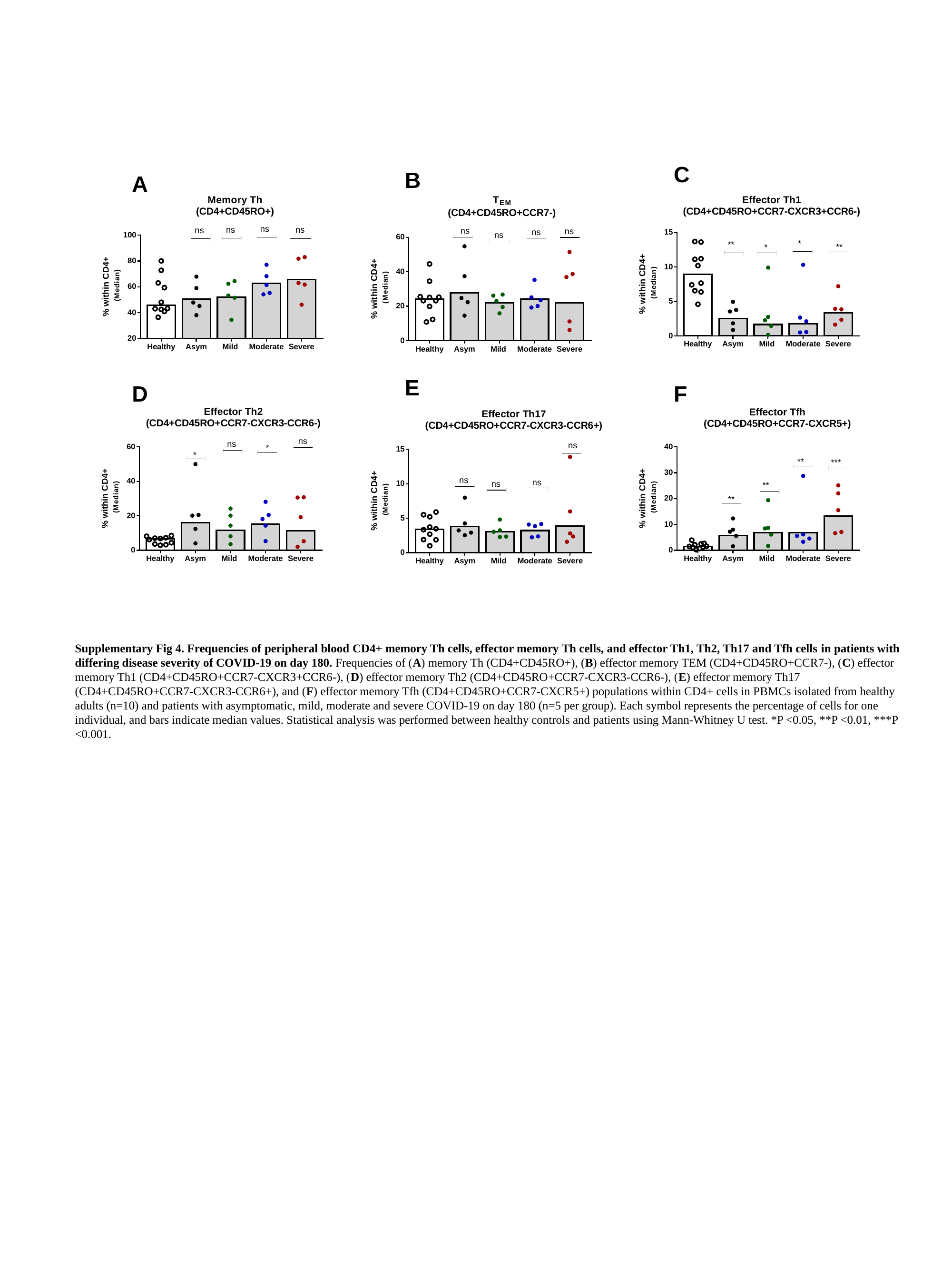

Supplementary Fig 4. Frequencies of peripheral blood CD4+ memory Th cells, effector memory Th cells, and effector Th1, Th2, Th17 and Tfh cells in patients with differing disease severity of COVID-19 on day 180. Frequencies of (A) memory Th (CD4+CD45RO+), (B) effector memory TEM (CD4+CD45RO+CCR7-), (C) effector memory Th1 (CD4+CD45RO+CCR7-CXCR3+CCR6-), (D) effector memory Th2 (CD4+CD45RO+CCR7-CXCR3-CCR6-), (E) effector memory Th17 (CD4+CD45RO+CCR7-CXCR3-CCR6+), and (F) effector memory Tfh (CD4+CD45RO+CCR7-CXCR5+) populations within CD4+ cells in PBMCs isolated from healthy adults (n=10) and patients with asymptomatic, mild, moderate and severe COVID-19 on day 180 (n=5 per group). Each symbol represents the percentage of cells for one individual, and bars indicate median values. Statistical analysis was performed between healthy controls and patients using Mann-Whitney U test. *P <0.05, **P <0.01, ***P <0.001.

## Slide 5
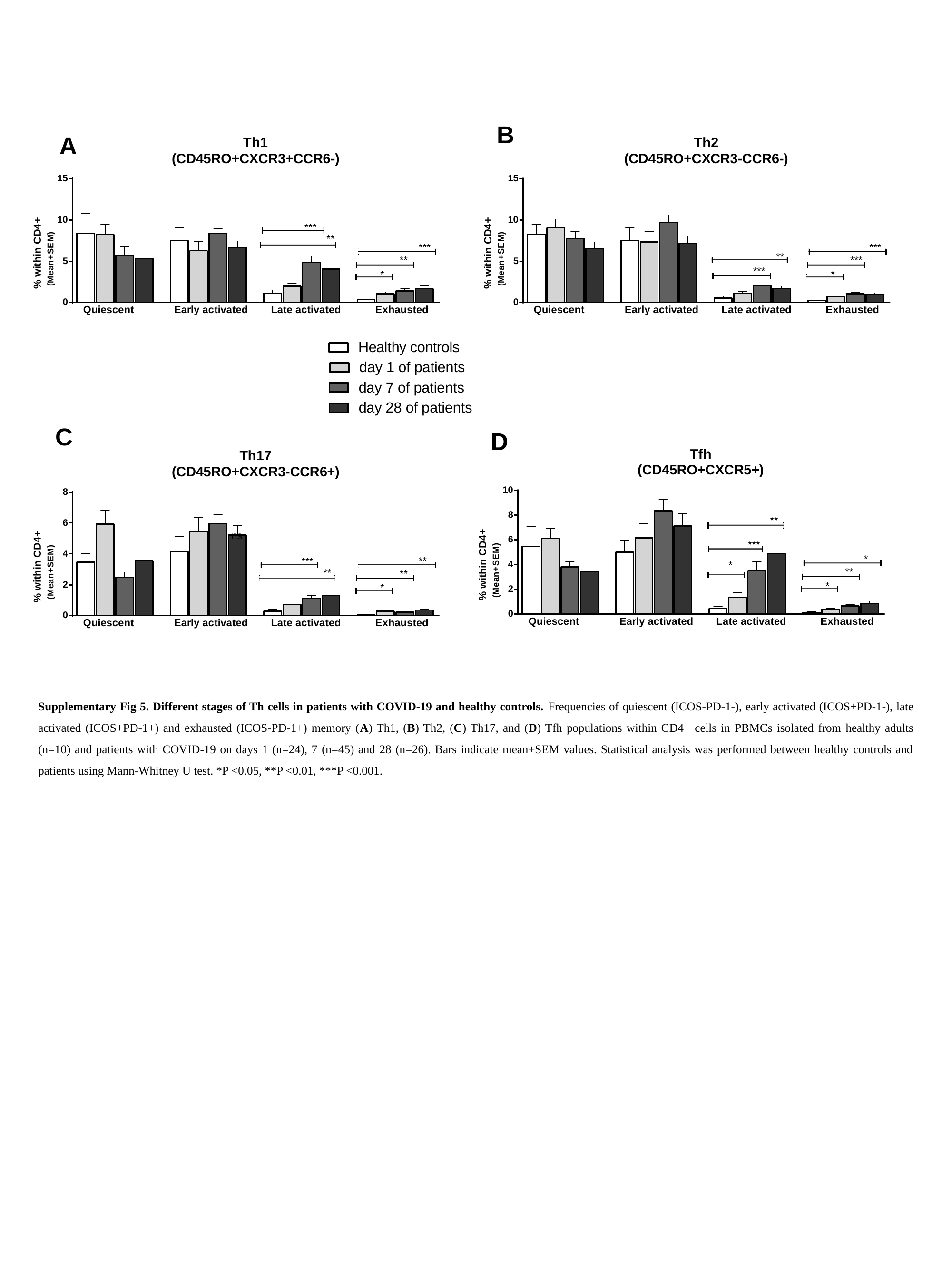

Supplementary Fig 5. Different stages of Th cells in patients with COVID-19 and healthy controls. Frequencies of quiescent (ICOS-PD-1-), early activated (ICOS+PD-1-), late activated (ICOS+PD-1+) and exhausted (ICOS-PD-1+) memory (A) Th1, (B) Th2, (C) Th17, and (D) Tfh populations within CD4+ cells in PBMCs isolated from healthy adults (n=10) and patients with COVID-19 on days 1 (n=24), 7 (n=45) and 28 (n=26). Bars indicate mean+SEM values. Statistical analysis was performed between healthy controls and patients using Mann-Whitney U test. *P <0.05, **P <0.01, ***P <0.001.
